# Taxonomic revision proves *Trachusa pubescens* (Morawitz, 1872) sensu lato to be a complex of allopatric and sympatric species in South-Eastern Europe and Western Asia (Hymenoptera, Apoidea, Anthidiini)

**DOI:** 10.3897/zookeys.764.24581

**Published:** 2018-06-05

**Authors:** Max Kasparek

**Affiliations:** 1 Mönchhofstr. 16, 69120 Heidelberg, Germany

**Keywords:** Anthidiini, *Anthidium*, Apoidea, Balkans, Middle East, new species, new status, Palaearctic region, taxonomy, *Trachusa*

## Abstract

*Trachusa pubescens* (Morawitz, 1872) s. l. has a distribution extending from south-eastern Europe over Anatolia and the Caucasus to Iran and Turkmenistan, and was formerly regarded as a species with high intraspecific variation. By means of an examination of 208 specimens from all parts of the distribution area, covering structural features of the head (mandibles, clypeus), the apical terga and the genitalia, the colouration pattern as well as a morphometric analysis of 26 body measurements with multivariate statistical methods (Principal Component Analysis, Discriminant Analysis), it was possible to assign the material to five species of which two are new to science (*Trachusa balcanica* **sp. n.** and *T. hakkariensis* **sp. n.**). Two taxa which had previously been described as “variations” or subspecies are elevated to species rank: *T. verhoeffi* (Mavromoustakis, 1955), **stat. n.** and *T. maxima* (Friese, 1931), **stat. n.** Additionally, some populations can be distinguished by their colouration pattern or by subtle differences in size or body shape, but these features are apparently of no taxonomic significance at the species level. *Trachusa balcanica* sp. n. and *T. verhoeffi* have distribution areas which do not overlap with any of the other members of the species group and can thus be characterised as allospecies. By contrast, the distribution areas of the other three species, *T. pubescens*, *T. maxima* and *T. hakkariensis* sp. n., overlap to a certain extent and they co-exist at least to some degree in sympatry. While they have been found in the same region, they have so far never been found together at exactly the same location and it is suggested that species divergence occurred in parallel with ecological differentiation. Niche partitioning such as flower preferences is a mechanism which may be invoked to explain this. Some specimens with intermediate characters were found, particularly in contact zones, and it is thought that some hybridisation may occur. A partly melanistic individual of *T. balcanica* **sp. n.** was found, which is probably the first described melanistic individual in the tribe Anthidiini.

## Introduction

*Trachusa pubescens* (Morawitz, 1872) is a relatively large species of bee belonging to the tribe Anthidiini, originally described in the genus *Anthidium* and with a characteristic wasp-like black-yellow colouration pattern. It was first described from the Caucasus and has since been found in south-eastern Europe, Anatolia, Iran and the Levantine countries. Several “varieties” and subspecies have been described (Morawitz 1872; Mocsáry 1879, 1884; Friese 1931; Mavromoustakis 1955). Pasteels (1969) wrote about the “*pubescens* group” comprising three species, while Warncke (1980) considered *pubescens* as the only species with two subspecies. This opinion has been shared in various recent publications (e.g. Ascher and Pickering 2016, Kuhlmann 2016, Grace 2010, Kasparek 2017a).

While males of *Trachusa pubescens* s. l. can easily be identified by their characteristic tripod-shaped apical tergum which is unique in anthidiine bees (Warncke 1980, Kasparek 2017a), a high degree of variation in size, shape, and colouration makes it difficult to decide what is intraspecific variation and what is of taxonomic significance for delimiting taxa. In addition to a classical approach relying on structural and colouration features, I thus also used a morphometric analysis of a series of body measurements. Multivariate statistical methods such as Principal Component Analysis and Discriminant Analysis, which may reveal possible subtle morphometric differences in three-dimensional morphological structures, are still rarely used in the taxonomy of solitary bees but have recently been introduced into anthidiine taxonomy (Kasparek 2017b). For this study I worked with a relatively large number of specimens from all parts of the distribution range, assembled from several public and private collections.

Taking into account the fact that the West Palaearctic *Trachusa* bees include further instances of complex and taxonomically little understood forms (e.g. *Trachusa interrupta* s. l.), the case of *T. pubescens* s. l. may provide a model for use in other groups.

## Materials and methods

Altogether 208 specimens (60 females, 148 males) of *T. pubescens* s. l. have been examined from 17 countries (Table 1). Material deposited in the Senckenberg Deutsches Entomologisches Institut, Müncheberg (Germany), Senckenberg Museum Frankfurt (Germany), Oberösterreichisches Landesmuseum, Linz (Austria), Snow Entomological Collection, University of Kansas, Kansas (United States of America), Ivan I. Schmalhausen Institute of Zoology of the National Academy of Sciences, Kiev (Ukraine), and Museum für Naturkunde Berlin (Germany) as well as in the collection of George A. Mavromoustakis at the Ministry of Agriculture, Nicosia (Cyprus), and the private collections of Maximilian Schwarz, Ansfelden (Austria), Werner Arens, Bad Hersfeld (Germany), Alireza Monferad, Yasouj (Iran) and the author were examined. Additionally, material deposited in the Hungarian Natural History Museum, Budapest, was used for the distributional analysis, and type and other material deposited in the Zoological Institute of the Russian Academy of Sciences, St. Petersburg (Russia) was examined by means of photographs.

**Table 1.** Material of *Trachusa pubescens* s. l. examined from the countries in the distribution area.

	Female	Male	Total
Armenia	0	6	6
Bulgaria	2	2	4
Greece	9	15	24
Hungary	2	5	7
Iran	4	6	10
Israel	2	3	5
Jordan	0	2	2
Lebanon	0	2	2
Macedonia (Former Yugoslav Republic)	1	3	4
Palestine, State of	0	1	1
Romania	1	0	1
Russia	0	2	2
Serbia	2	4	6
Syria	1	0	1
Turkey	34	81	115
Turkmenistan	0	1	1
Ukraine	2	15	17
**TOTAL**	**60**	**148**	**208**

*Clustering of the material*. The material was grouped into Operational Units (OU) defined on the basis of morphological features and/or geographic occurrences. Due to sympatric occurrences, OUs are not necessarily identical with geographically defined populations.

*Species concept.* As *T. pubescens* s. l. exhibits a high degree of variation, a clear taxonomic concept is required which enables a decision to be made as to whether an OU corresponds to the rank of a species or subspecies. In principal, I follow the “Guidelines for assigning species rank” developed by the British Ornithologists’ Union (Helbig et al. 2002). Two OUs are considered distinct species if they maintain phenotypic integrity in sympatry. Geographic replacement species are considered as distinct species if their contact zone consists of an abrupt transition with little phenotypic intergradation. Subtle morphological differences in size and/or shape, as revealed for example by morphometric analysis (Discriminant Analysis), are not considered as being sufficient for assigning these populations to different taxa if these differences are not further supported by colour or other features. Two forms separated by a cline are considered as belonging to the same species as gene flow is limited only through isolation by distance, not as a result of an intrinsic barrier. Following Mayr (1975), clines are therefore not given nomenclatural recognition. This principle was also applied in cases where the available material did not allow a decision whether there is a continuous or a stepped cline (and hence whether possibly different species are involved).

*Distinguishing characters.* The morphological characters which are presented here for distinguishing the members of the *pubescens* species complex mostly refer to males, with the shape of the clypeus and mandibles and of the two apical abdominal segments being the most relevant ones. Females are not only less differentiated, but they are also rarer than males in collections. Of 208 specimens examined, 60 (28.8%) are females and not all of them could be unambiguously attributed to one of the species. The identification key is therefore confined to males only. The diagnosis of the members of the species complex presented below is confined to distinguishing characters. Within the species descriptions, emphasis is placed on individual variation and on differences between populations.

**Figure 1.**
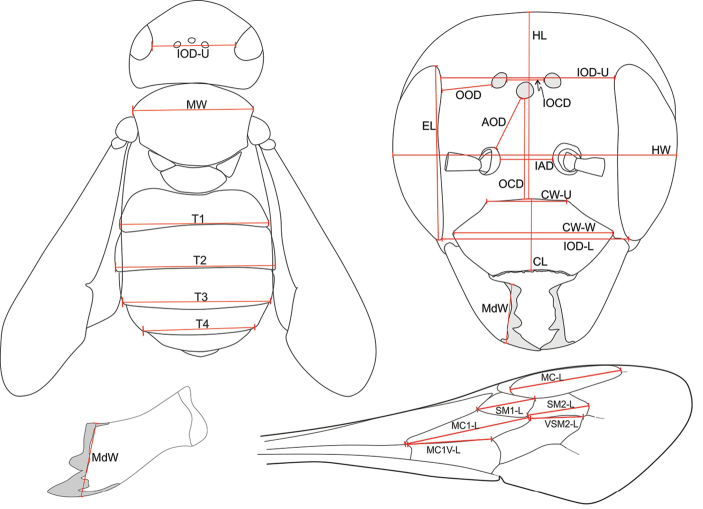
Measurements taken for the morphometric analysis. Note that the measurements were taken from positions in which both points of reference become equidistant from the lens of the microscope. This is not necessarily the perspective shown in the drawing.

*Measurements.* In order to discover morphological differences, 26 morphological measurements were taken (Fig. 1, Table 2). Measurements were taken with an ocular micrometer placed in the eye-piece of a Stereozoom microscope at different magnifications between 1.0x and 4.5x. The divisions of the ocular micrometer were converted to millimetres (mm) with the help of an objective micrometer. Measurements related to the ocelli and the clypeus and mandibles were photographed and measured with TSView7 (version 7.3.1.7) software. For obtaining exact measurements, it is crucial that the points of reference are equidistant from the lens of the microscope. This is challenging because of the three-dimensional shape of the specimens and needs some experience on the part of the observer. In order to reduce errors, many of the measurements were taken twice. Furthermore, all measurements were taken by the same person and with the same instruments. Due to sexual dimorphism and the lower number of females available, the morphometric analysis was confined to males. Terminology follows Michener (2007).

**Table 2.** List of measurements taken from the body of *Trachusa pubescens* for the morphometric analysis.

**Head**	
HL	Head length (from upper edge of clypeus to preoccipital ridge)
HW	Head width (at height of antennal sockets)
CW-W	Clypeus width (at widest point)
CW-U	Clypeus width (at upper end)
CL	Clypeus length (along middle line; excluding the dark crenulated apical margin)
EL	Eye length
IOD-L	Lower interocular distance (at widest point of clypeus)
IOD-U	Upper interocular distance (at the height of the centres of the posterior ocelli)
OCD	Ocellar-clypeal distance (from upper edge of clypeus to outer margin of anterior ocellus)
IOCD	Interocellar distance (from inner margins of posterior ocelli)
IAD	Interantennal distance
OOD	Ocello-ocular distance (shortest distance, which can be found after some rotation)
AOD	Antennocellar distance (from antennal socket to margin of anterior ocellus)
MdW	Mandible width
**Mesonotum**	
MW	Width of mesonotum (at widest point)
**Metanotum**	
T1	Width of T1 (at distal margin)
T2	Width of T2 (at distal margin)
T3	Width of T3 (at distal margin)
T4	Width of T4 (at distal margin)
**Wing**	
MC-L	Length of marginal cell (from inner proximal edge to inner distal edge)
SM1-L	Length of 1^st^ submarginal cell
SM2-L	Length of 2^nd^ submarginal cell
VSM2-L	Length of vein of 2^nd^ submarginal cell
MC1-L	Length of 1^st^ medial cell
MC1V-L	Length of vein of 1^st^ medial cell

*Statistical treatment.* Multivariate statistical procedures were applied for analysing morphometric data. In principal, the analysis was conducted in two steps: First, an assessment was made as to whether the specimens analysed could be grouped in certain clusters. This was done with a Principal Component Analysis (PCA), which was used here as a tool for exploratory data analysis. In a second step, a Discriminant Function Analysis (DFA) (= Canonical Variate Analysis, CVA) was performed to determine whether a set of body measurements is effective in predicting category membership. An important difference between PCA und DFA is that PCA examines datasets where membership of a particular Operational Unit (OU) is not known, while DFA classifies datasets whose OU has been previously determined. Based on the assumption that all measurements are normally distributed, the parametric one-way ANOVA (Analysis of Variance) was used to determine whether there are statistically significant differences between the means of three or more OUs. In order to find out which OU means (compared with each other) are different, the Tukey test was applied. The test compares all possible pairs of means.

The statistical tests were performed with XLSTAT Version 2015.6.0123990, which is a statistical software package for Microsoft Excel, and PAST (PAleontological STatistics), Version 3.16 (2017) (Hammer et al. 2001).

### Abbreviations and Acronyms

**DIE** Senckenberg Deutsches Entomologisches Institut (Germany)

**OLL** Oberösterreichisches Landesmuseum Linz (Austria)

**SEMC** Snow Entomological Collection, University of Kansas, Kansas (United States of America)

**SIZK** Ivan I. Schmalhausen Institute of Zoology of the National Academy of Sciences, Kiev (Ukraine)

**SMF** Senckenberg Museum Frankfurt (Germany)

**ZISP** Zoological Institute, Russian Academy of Sciences, St. Petersburg (Russia)

**ZMB** Museum für Naturkunde Berlin (Germany)

**cAM** Collection Alireza Monferad, Yasouj (Iran)

**cMK** Collection Max Kasparek, Heidelberg (Germany)

**cMS** Collection Maximilian Schwarz, Ansfelden (Austria)

**cMAV** Collection George A. Mavromoustakis, Ministry of Agriculture, Nicosia (Cyprus)

**cWA** Collection Werner Arens, Bad Hersfeld (Germany)

**OU** Operational Unit

**S1, S2**, etc. first, second, etc., metasomal sternum

**T1, T2**, etc. first, second, etc., metasomal tergum

## Results

### *Trachusa* Panzer, 1804

#### Trachusa (Archianthidium) Mavromoustakis, 1939

##### The *Trachusa pubescens* species complex

*Trachusa pubescens* s.l. has been assigned to the subgenus Trachusa (Archianthidium), which to date comprises six species (Kasparek 2017a, 2017b) and is characterised by the position in which the second recurrent vein enters the second submarginal cell and the presence of a projection on tergum T7 of the male (Michener 2007). A series of characters support the view that the genus *Trachusa* is sister to the rest of Anthidiini (Litman et al. 2016).

Descriptions of *T. pubescens* s. l. are given by Morawitz (1872), Friese (1931), Mavromoustakis (1955), Pasteels (1969), Kasparek (2017a), and others. The males of all members of the species complex are characterised by a typical tripod-shaped T7, and the females by broad black mandibles in combination with an entirely yellow clypeus and yellow markings in the genal area. *Trachusa pubescens* s. l. is known as being highly variable. When Morawitz (1872) described the species from the Caucasus, he mentioned three “varieties” according to differences in the colouration of the scutum (var. a), scutellum (var. b) and T6 (var. c). Mocsáry (1879) added two more “variations” from Hungary: “Variat. a” with entirely black antennal scape, and “Variat. b” without a yellow spot on mesepisternum (mesopleurum). Friese (1931) described the “var. maximum” from Turkey, which is twice the size of specimens from Hungary but with only minor structural differences. Mavromoustakis (1955) added the subspecies *verhoeffi* from the Levant, with a distinct colouration and some differences in the form of T6. Pasteels (1969) recognised [*Archianthidium*] *pubescens*, *maximum* and *verhoeffi* as valid species, while Warncke (1980) synonymised *maximum* with the nominate form and recognised *verhoeffi* as the only valid subspecies in addition to the nominate subspecies. This opinion has been shared in various recent publications including the Discover Life web presentation (Ascher and Pickering 2016), the Checklist of the Western Palaearctic Bees (Kuhlmann 2016), a biogeographic study of the bees of the Eastern Mediterranean and Near East (Grace 2010) and a recent review of the genus by Kasparek (2017a).

**Figure 2.**
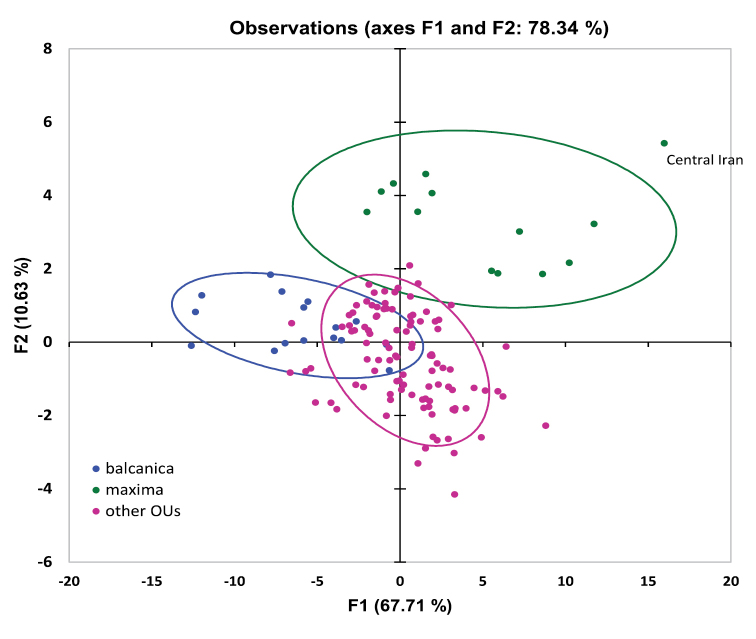
Principal Component Analysis (PCA) of 26 morphometric variables of males of *Trachusa pubescens* s. l. (N = 132). Individuals subsequently assigned to *T. balcanica* sp. n. and *T. maxima* are shown here in a different colour. All other OUs are shown here in the same colour. One specimen from central Iran is significantly different from all other specimens of the group. The confidence ellipses show a confidence interval of 80%.

For an initial characterisation of the *T. pubescens* complex, a Principal Component Analysis (PCA) was conducted based on the correlation matrix of 26 morphometric data (Fig. 2). The first two eigenvalues represent 78.34% of the initial variability of the data, and this relatively high value shows that the first two factors are a good quality projection of the initial multi-dimensional table. All variables throughout have factor loadings >0.6 in the first component F1, while variables related to the wing also have high factor loadings in component F2. The PCA further shows a clear separation of two groups of individuals that can be identified as the *balcanica* OU and the *maxima* OU (Fig. 2). This can be taken as the first evidence that these differences are of taxonomic significance. Within the *maxima* OU, one specimen from central Iran is different from the others in both its F1 and F2 values and needs to be further examined (see below).

**Figure 3.**
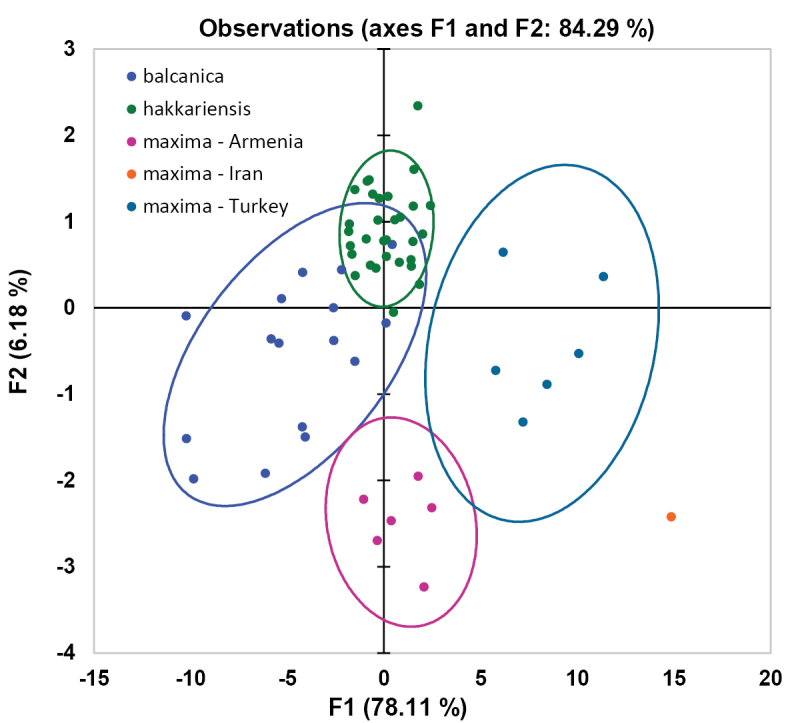
Principal Component Analysis (PCA) of 26 morphometric characters (males) of those OUs of the *Trachusa pubescens* complex whose members are characterised by an emarginate clypeus and subacute lateral projections on T6.

In order to refine the results, another PCA was carried out on the morphometric data of only those specimens which are characterised by a combination of an emarginate clypeus with subacute lateral projections of T6: the data clearly cluster in five groups (Fig. 3), and these groups reflect the taxa *balcanica* sp. n., *maxima*, and *hakkariensis* sp. n. Within *T. maxima*, the OUs from Turkey, Armenia and Iran can be distinguished (for Iran, only one specimen is available). As the PCA clusters the specimens without knowing their group membership, a DA was conducted after having assigned the material to these three taxa. Fig. 16 shows that all specimens are well separated on the species level without overlapping cases, while *maxima* from Turkey cannot be distinguished from *maxima* from Armenia. A confusion matrix shows that all specimens (100%) are assigned correctly to the three taxa *balcanica* sp. n., *maxima*, and *hakkariensis* sp. n.

The size of the mandible shows considerable variation, and the average values were compared for the various OUs. Mandible width is on average greater in *T. maxima* than in all other groups of the complex (Figs 5, 14). All pairwise comparisons of the mandible width of the five species were tested with the parametric Tukey’s test (based on the assumption of normally distributed data) and it was shown that *T. maxima* has highly significantly (*p*<0.001) wider mandibles than all other species (Table 3), but that the other species do not show significant differences among themselves (*p*>0.05). The large mandible is therefore regarded as a unique character of *T. maxima*.

**Figure 4.**
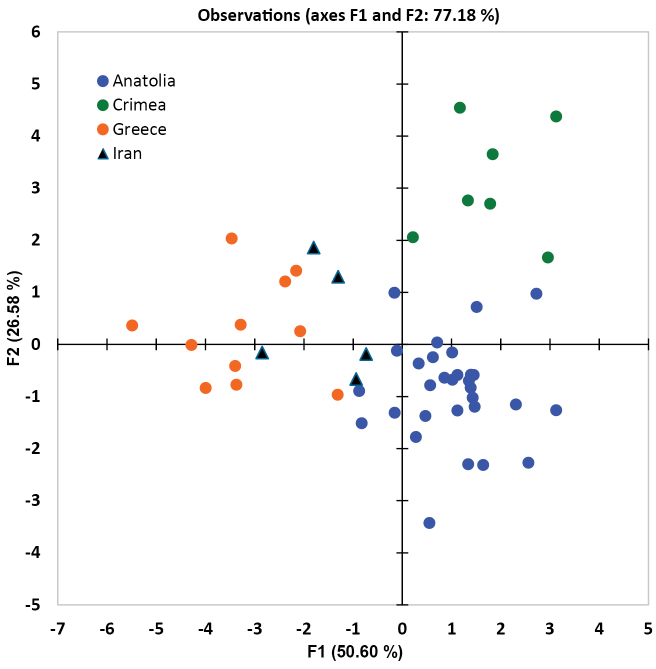
Results of a Discriminant Analysis of the Operational Units (OU) assigned to *Trachusa pubescens* s. str. based on 26 morphometric characters of the male.

**Figure 5.**
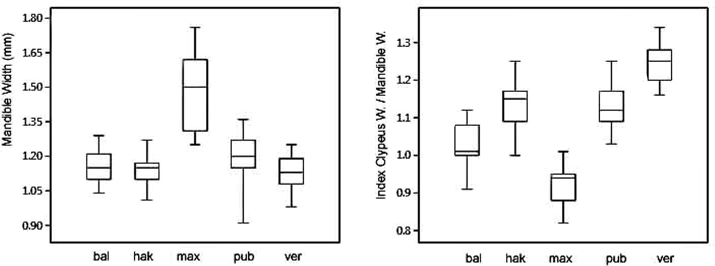
Mandible width (left) and index clypeus length / mandible width in males of the five species of the *Trachusa pubescens* complex. Abbreviations: bal = *T. balcanica* sp. n.; hak = *T. hakkariensis* sp. n.; max = *T. maxima*; pub = *T. pubescens*; ver = *T. verhoeffi*.

**Table 3.** Comparison of the mandible width and the index clypeus length / mandible width among the five species of the *Trachusa pubescens* complex. The table gives the significance values for differences according to Tukey’s pairwise test. The upper part above the diagonal (grey cells) gives the p values for mandible width, the lower part the values for the index.

	*balcanica*	*hakkariensis*	*maxima*	*pubescens*	*verhoeffi*
*balcanica*	–	0.977	**<0.001**	0.501	0.843
*hakkariensis*	**<0.001**	–	**<0.001**	0.185	0.992
*maxima*	**<0.001**	**<0.001**	–	**<0.001**	**<0.001**
*pubescens*	**<0.001**	0.999	**<0.001**	–	0.066
*verhoeffi*	**<0.001**	**<0.001**	**<0.001**	**<0.001**	–

As *T. maxima* is also larger than the other species, a further test investigated how mandible size relates to clypeus size. In this way, the possibility that the large mandible size is merely a consequence of the large body size can be excluded. For this purpose, a pairwise comparison among all five species was applied to the index clypeus length / mandible width (Table 3). *Trachusa maxima* has again the relatively biggest mandible as compared to all other species. However, significant differences (*p*<0.001) are also found among all the other species with the exception of the pair *T. hakkariensis* sp. n. / *T. pubescens* whose index did not differ at a significant level (*p*>0.05).

**Figure 6.**
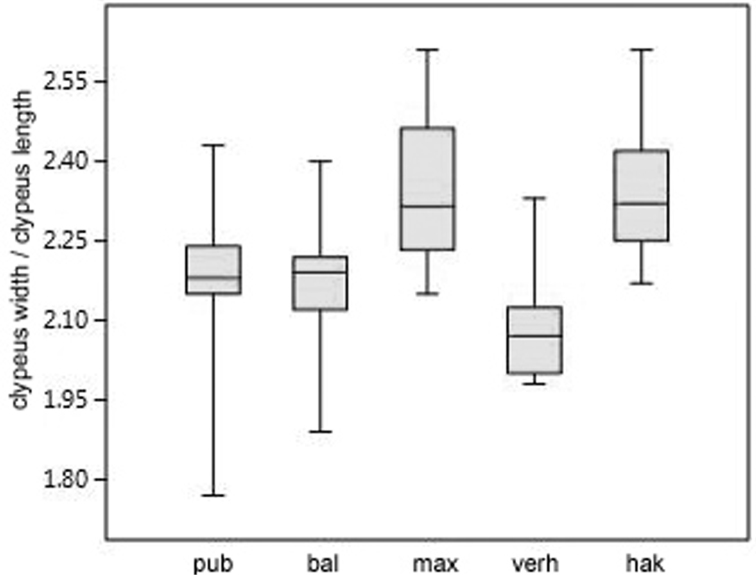
Comparison of the clypeus index (clypeus width / clypeus length) in males of the five species of the *Trachusa pubescens* complex. Abbreviations: bal = *T. balcanica* sp. n.; hak = *T. hakkariensis* sp. n.; max = *T. maxima*; pub = *T. pubescens*; ver = *T. verhoeffi*.

A further test investigated whether the members of the *Trachusa pubescens* complex show differences in the shape of the clypeus, and for this purpose the clypeus index (index clypeus width to clypeus length) was compared (Fig. 6). The results show that *T. maxima* and *T. hakkariensis* sp. n. have the relatively widest clypeus and that there are no significant differences between these two species (Table 4). *Trachusa balcanica* sp. n. and *T. pubescens* take a medium position with regard to the clypeus index, and again these two species do not differ significantly in this character. *Trachusa verhoeffi* has the relatively narrowest clypeus and it is significantly narrower than in all other species (Table 4).

**Figure 7.**
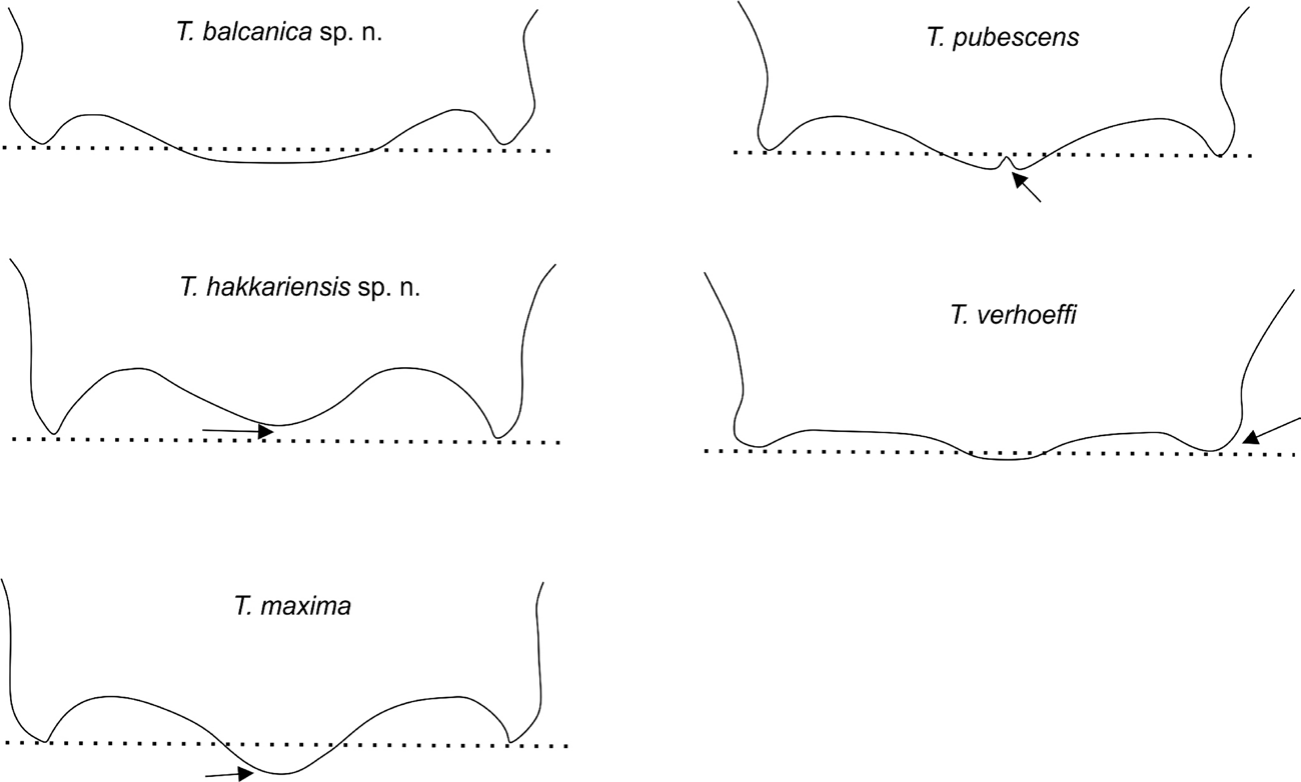
Dorsal view of the apical margin of T6 in the five species of the *Trachusa pubescens* complex. The dotted line is added to show the relative length of the median projection in relation to the lateral projections. Arrows indicate distinguishing features.

**Figure 8.**
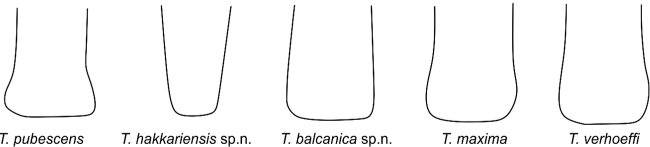
Dorsal view of the apex of the median projection of T7 in the five species of the *Trachusa pubescens* complex.

Considerable differences were found in the shape of the apical terga (T6 and T7) in the males of the five species (Figs 7, 8, 15), which are described under the species descriptions below.

**Table 4.** Comparison of the clypeus index (clypeus width / clypeus length) of the five species of the *Trachusa pubescens* complex. The table gives the significance values for differences according to Tukey’s pairwise test.

	*balcanica*	*hakkariensis*	*maxima*	*pubescens*	*verhoeffi*
*balcanica*	–	**<0.001**	**<0.001**	0.536	**<0.010**
*hakkariensis*		–	0.932	**<0.001**	**<0.001**
*maxima*			–	**<0.001**	**<0.001**
*pubescens*				–	**<0.001**
*verhoeffi*					–

**Figure 9.**
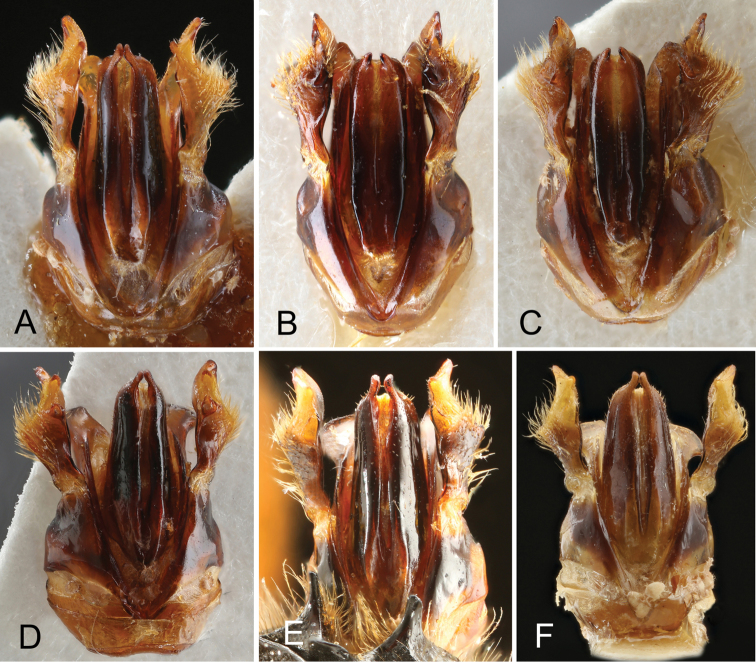
Male genitalia of the five species of the *Trachusa pubescens* complex. **A** *Trachusa balcanica* sp. n. (FYR of Macedonia) **B–C** *T. pubescens* (two different specimens from Greece) **D** *T. maxima* (Armenia) **E** *T. verhoeffi* (SW Turkey) **F** *T. verhoeffi* (Israel).

**Figure 10.**
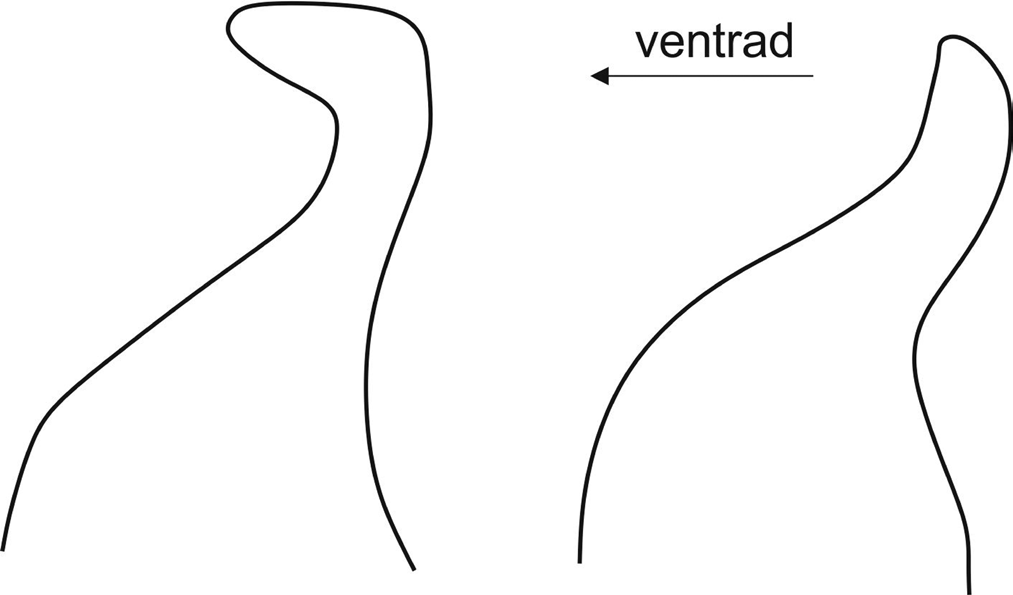
Apices of the penis valves in the *Trachusa pubescens* complex. Hook-shaped apices (left) are found in *Trachusa balcanica* sp. n., *T. maxima*, *T. hakkariensis* sp. n., and *T. verhoeffi*. The apices of the penis valves are straight only in *T. pubescens* (right).

The apices of the penis valves are almost straight and subacuminate in *T. pubescens* (genitalia of three specimens from Greece, one from Tatvan/Eastern Turkey, and one from Iran examined) while they are slightly elongated and bent ventrad hook-like in the other four species: *T. balcanica* sp. n. (one specimen examined), *T. maxima* (four specimens from Armenia examined), *T. hakkariensis* sp. n. (seven specimens examined), and *T. verhoeffi* (one specimen from Israel, one from Lebanon, and eight from SW Turkey examined) (Figs 9, 10).

###### Trachusa balcanica sp. n.

Taxon classificationAnimaliaHymenopteraMegachilidae

http://zoobank.org/AB35EA8B-470E-43BD-836C-ABD1F1AAFFDC

**Figure 11.**
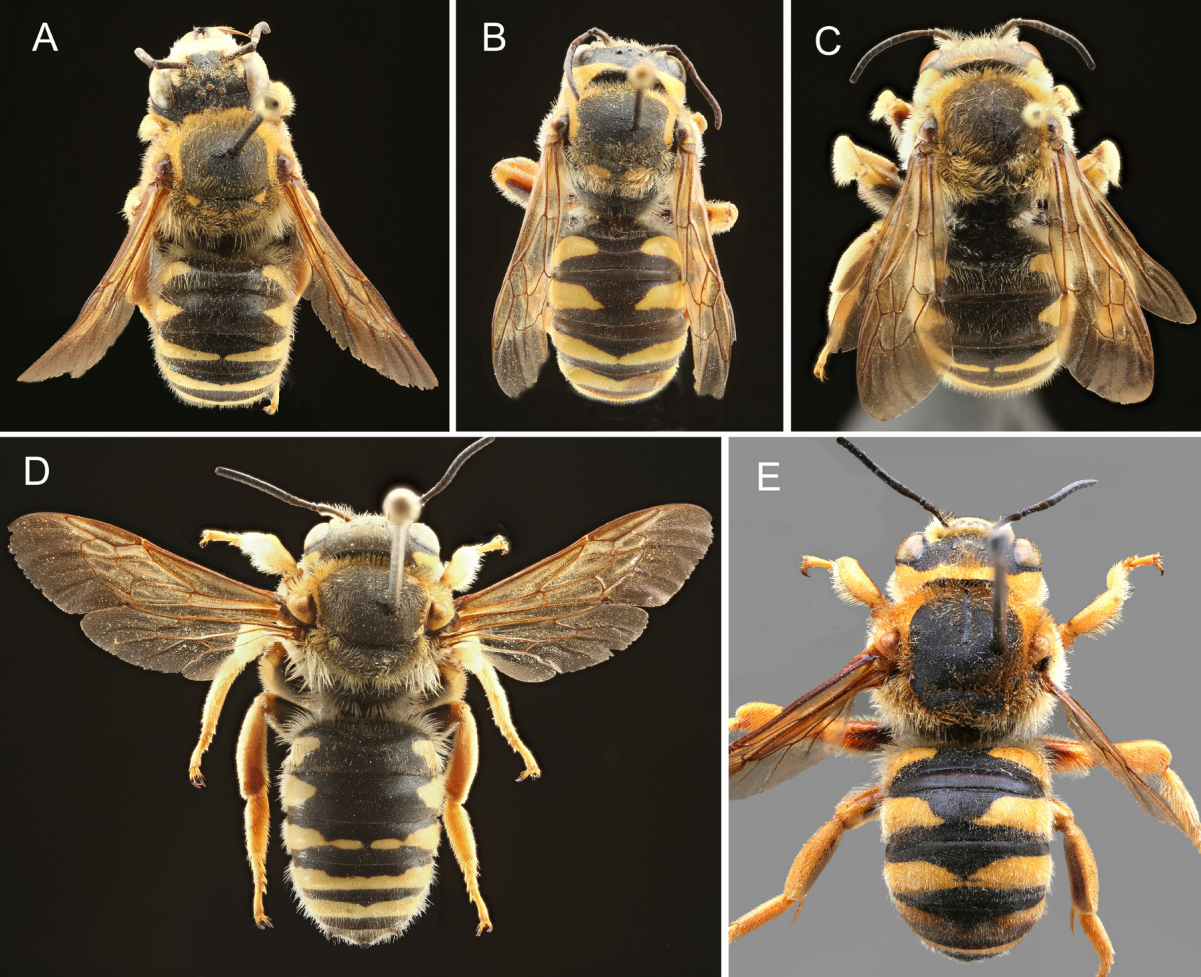
Males of the five species of the *Trachusa pubescens* complex in dorsal view: **A** *T. balcanica* sp. n. **B** *T. hakkariensis* sp. n. **C** *T. maxima* **D** *T. pubescens* **E** *T. verhoeffi*.

**Figure 12.**
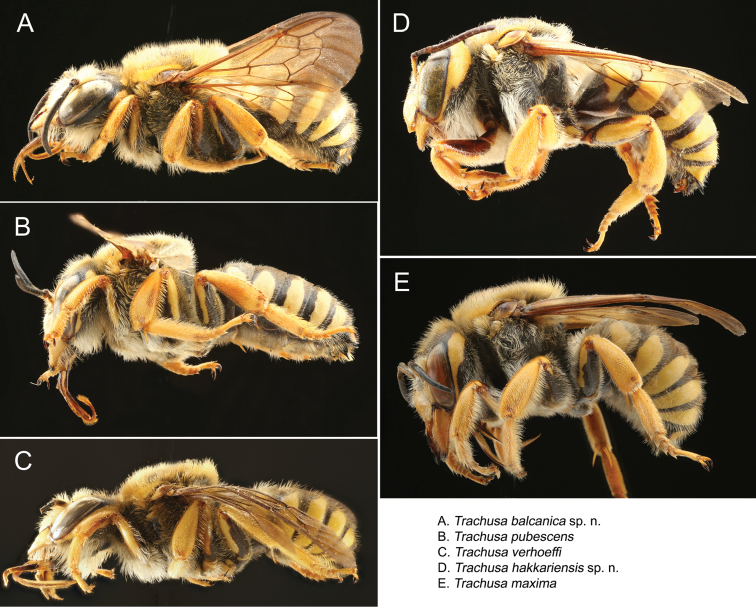
Males of the five species of the *Trachusa pubescens* complex in lateral view.

**Figure 13.**
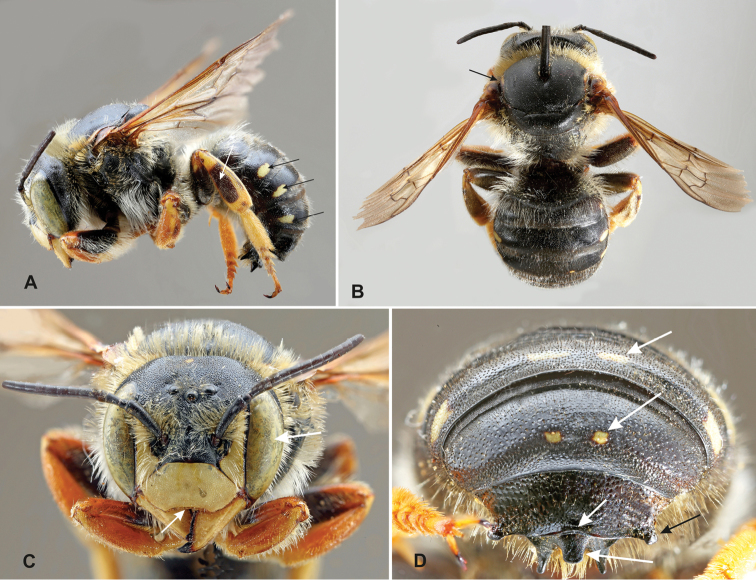
*Trachusa balcanica* sp. n., semi-melanistic individual from Greece.

**Figure 14.**
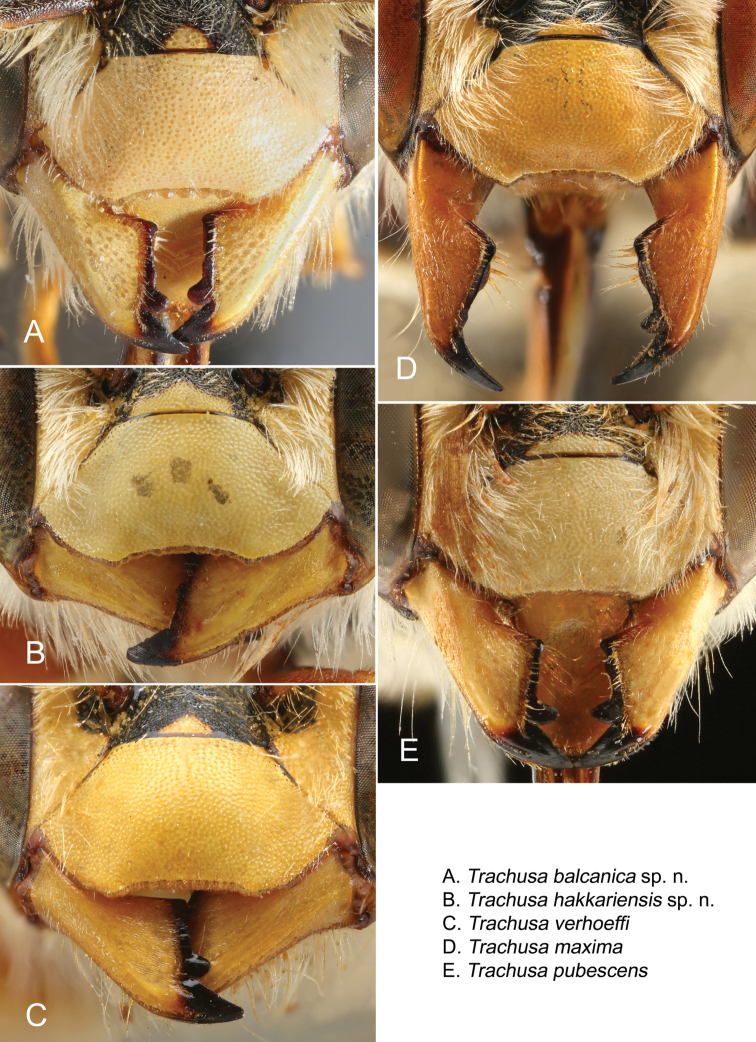
Clypeus and mandibles of the males of the five species of the *Trachusa pubescens* complex.

**Figure 15.**
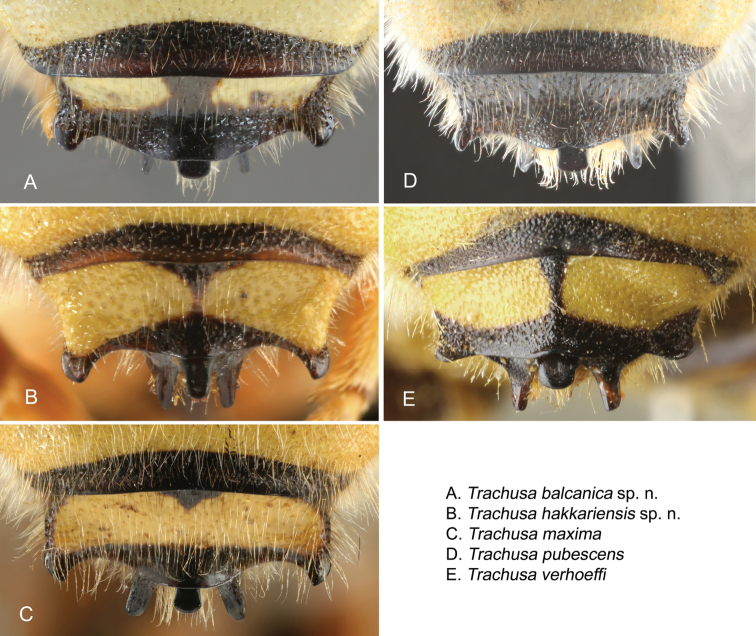
The two apical terga (T6–T7) of males of the five species of the *Trachusa pubescens* complex.

Figs 11 , 12 , 13 , 14 , 15

####### Material.

Holotype: Male. **BULGARIA**: Sandanski (Blagoevgrad Province, south-western Bulgaria), June 1972, K. Poláček leg. (cMS). – Paratype (1): **BULGARIA**: male, same location as holotype, 26.-31.5.1967, Kocourek leg. (cMS).

Further material examined (21): **BULGARIA**: 1♀, Sandanski, 26.–31.5.1967 (cMS); 1♀, ibid., 1.–8.06.1967 (cMS). **GREECE**: 1♀ 1♂, 35 km NE Kalambaka, 15.05.2005, J. Halada / M. Kadlecová leg. (cMS); 1♂, Hellas, Kastoria, Aposkepos (850 m), 06.vii.1967, J. Reinig leg. (SMF); 1♂, Koupaki (38°30'N, 22°01'E), northwestern part of Phocis, central Greece, 21.05.1990, H. Malicky leg. (cMS). **HUNGARY**: 2♂, Hungary, E. Frivaldski leg. (ZMB); 1♂, South Hungary, ex coll. Schmiedeknecht (ZMB); 1♀ 1♂, central Hungary, ex coll. Alfken (ZMB); 1♀ 1♂, Budapest, A. Mocsáry leg. (ZMB). **MACEDONIA** (Former Yugoslav Republic): 2♂, Prilep, 01.06.1968, K. Warncke leg. (OLL). **SERBIA**: 1♀, 1♂, Deliblat, 23.07.1886, H. Friese leg. (SMF); 1♀, 3♂, same data (ZMB).

Material not examined: The Hungarian Natural History Museum holds 11 female and 27 male specimens labelled as “*Trachusa pubescens*” which according to the collection localities can most probably be assigned to *T. balcanica*: Deliblat (Serbia), Budapest (Hungary), Grebenac (Serbia), Kecskemét (Hungary), Halas [=Kiskunhalas] (Hungary), Peszér [=Kunpeszér] (Hungary), Kecel (Hungary), and “Hungariae centralis”. Some males have no locality label at all.

####### Differential diagnosis.

The smallest species of the *T. pubescens* complex (mostly 13–16 mm versus mostly 16–20 mm). Males are separated from *T. pubescens*, the only other European species of the complex, by the conspicuously emarginate apex of the clypeus (almost straight in *T. pubescens* s. str.), with 8–11 small tubercles in the emargination (indistinguishable or hardly distinguishable tubercles in *T. pubescens*). *Trachusa balcanica* sp. n. shares this feature with the remaining members of the species group.

Both sexes have a yellow stripe on vertex, sometimes attenuated in the middle or reduced to small remnants. This stripe is absent in *T. pubescens* s. str. which also occurs on the Balkans. Yellow maculation in the genal area in *T. balcanica* sp. n. is usually confined to the upper half (usually one-third); in only two out of 23 males, the genal maculation extends slightly on to the lower half, but never reaches the lower end of the eye as in *T. hakkariensis* sp. n. and *T. maxima*, or extends over most of the genal area as in *T. pubescens* and *T. verhoeffi*. In eight females examined, the yellow maculation extends in three cases slightly onto the lower half but is confined to the upper half in the other cases.

T6 of males has a broad, usually rounded median projection; apex rounded or at most truncated but never emarginate as in *T. pubescens* (Figs 7, 15). Lateral projections subacute. Punctation of T6 finer and more scattered in comparison with *T. pubescens*. Median projection of T7 parallel-sided, apex truncated (Figs 8, 15).

Pubescence on thorax dense and relatively long; pubescence on vertex and dorsal side of mesosoma reddish brown. Pubescence in the other species of the complex is inclined to be dull white to yellow-brown, but this difference can only be seen when series of specimens are compared.

####### Variability.

One of the males examined from Greece shows a colouration pattern which is different from all other specimens: The integument is shining black (not dull black), the paraocular area yellow (yellow maculation not reaching top of eye); one very small yellow spot on vertex and narrow yellow remnants of the anterolateral L-shaped band on the scutum; one small yellow lateral spot on each side of T1–T4, and additionally two small yellow median spots on T3 and T4 (no bands). While all yellow colouration is thus much reduced on the body, this specimen shows elongate light brown stripes on fore- and middle tibiae and one dark brown stripe on each hind tibia. While this colour pattern is entirely different from all other specimens examined in the *T. pubescens* complex, the size, the form of the clypeus, and the shape of T6 and T7 are in conformity with *T. balcanica* sp. n., and it is thought that this specimen belongs to this species.

####### Derivatio nominis.

The name is derived from the Balkans.

####### Distribution.

The distribution area of *Trachusa balcanica* sp. n. extends from central Hungary (Budapest) in the north over central Serbia to western Bulgaria. In the southwestern part of its range, it extends over Macedonia to northern and central Greece. Countries of occurrence are: Bulgaria, Greece, Hungary, Macedonia (F.Y.R.), and Serbia. Records from Romania (a female from Tulcea, 1895, J. J. Mann leg., cMAV, and a female listed by Calefariu 2017) and Moldova (Stratan and Andreev 2015) could not be examined, or not to the extent necessary to allow an unambiguous assignment to this species (*T. pubescens*?).

####### Flower preference.

Mocsáry (1884) found the species in central Hungary in July 1878 abundantly at *Stachys germanica*. Friese (1898) collected it at *Genista* in Serbia.

####### Remarks.

On 01.06.1965 and Prilep, FYR Macedonia, K. Warncke collected three males and a female of *T. pubescens* s. l., apparently together. While two males can be unambiguously attributed to *T. balcanica* sp. n. (shape of clypeus, in one male also shape of genitalia examined), the third male has the apical margin of the clypeus as in *T. pubescens* s. str. and was attributed to that species.

###### Trachusa hakkariensis sp. n.

Taxon classificationAnimaliaHymenopteraMegachilidae

http://zoobank.org/0BB022FC-2246-4427-830C-F0946B672D72

Figs 11 , 12 , 14 , 15

####### Material.

Holotype: Male. **TURKEY**: Hakkâri province: Cilo Dağı (W Serpil), 1800 m, 8.08.1982, K. Warncke leg. (OLL). Paratypes: 9♂, same data as holotype (OLL).

Other material: **TURKEY**: 14♂, Hakkâri province: Cilo Dağı (W Serpil), 1800 m, 8.08.1982, K. Warncke leg. (OLL); 1♂, Hakkâri province: Cilo Dağı (2000 m), M. Kühbandner leg. (cMS); 2♂, Van province: Gevaş, 2.07.2000, Ma. Halada leg. (cMS); 1♂, Malatya province: 15 km E Malatya, 27.06.2000, Ma. Halada leg.; 1♀, 3♂, Hakkâri province: 10 km NE Dağlıca (= Oramar) (1700 m), 29.06.1985, M. Schwarz leg. (cMS).

####### Differential diagnosis.

Males are similar in colouration to *T. maxima* but are clearly distinguished by the size of their mandibles (not enlarged as in *T. maxima*), the median projection of T6 which does not project beyond the lateral projections, and the on average smaller body size (16–18 mm versus 17–19 mm). In a few individuals the apex of the median projection of T6 is truncated (but never emarginate). The median projection of T7 normally tapers apically (usually parallel-sided in *T. maxima*). In the mandible index (clypeus length / mandible width), *T. hakkariensis* sp. n. resembles *T. pubescens* s. str. The average value is statistically significantly lower than *in T. verhoeffi* and higher than in *T. balcanica* sp. n. and *T. maxima* (Table 3).

*Trachusa hakkariensis* sp. n. has a shallowly emarginate clypeus with a crenulated apical margin. It shares this character with *T. balcanica* sp. n., *T. maxima*, and *T. verhoeffi*. By contrast, *T. pubescens* has a straight margin.

####### Derivatio nominis.

Named after the Turkish province of Hakkâri, where the type locality of the new species is located.

####### Distribution.

The species is known from eastern and south-eastern Turkey; records are available from the provinces of Hakkâri, Van and Malatya. The distribution areas thus overlap with *T. pubescens*, although these two species have never been found at the same location. The distribution areas of *T. hakkariensis* sp. n. and *T. maxima* are subcontiguous.

####### Flower preference.

No information available.

###### Trachusa maxima

Taxon classificationAnimaliaHymenopteraMegachilidae

(Friese, 1931) stat. n.

Figs 11 , 12 , 14 , 15

Anthidium pubescens var. maximum Friese, 1931: 37–38.

####### Note.

Warncke (1980) believed that Anthidium pubescens var. maximum Friese, 1931 was synonymous with the nominate *Anthidium pubescens* with some more yellow colouration and longer lateral projections of T6.

####### Material.

Holotype: Male. **TURKEY**: “Asia Minor Taurus pisid., 1928 / Type / *Anthidium pubescens* v. *maximum* Friese det. 1925 / Lectotype *Anthidium maximum* Männchen Friese 1931 (nec 1922) det. v. d. Zanden 1994” [see remark on collection locality below].

Other material: **ARMENIA**: 6♂, Khosrov State Reserve, 10.06.2017, M. Kasparek leg. (at *Phlomis*) (cMK). **IRAN**: 1♂, prov. Esfahan (50 km SW of Daran, pass betw. Aragol and Cohrud vill., 2800 m) 32.49.47N, 50.14.89E, 11.07.2001, M. Kalabza leg. (cMS). **TURKEY**: 1♂, Pisidian Taurus (“Pisidischer Taurus”), July 1928 (“*Archianthidium pubescens maximus* Fr. det. Mavromoustakis / lectoparatype *Anthidium maximum* Friese 1931 (nec 1921) det. v. d. Zanden 1994 / *Trachusa maximum* (Friese) det. v. d. Zanden 1994“) (cMAV); 2♂, Adıyaman, Kuyucak env., 10.06.2001, M. Snizek leg. (cMS); 1♂, Maraş-Afsin, 30.06.1984, K. Warncke leg. (OLL); 1♂, Sille near Konya, 9.–17.6.1975, J. Heinrich leg., J. Heinrich det. 1977 (SMF); 1♂, Ankara, 09.06.1934, H. Noack leg. (“*Anthidium laticeps* Alfken det. 1934 / *Archianthidium maximum* Friese J. Pasteels det. 1969”) (SMF).

####### Differential diagnosis.

The largest species of the *Trachusa pubescens* complex (17–20 mm versus 13–18 mm). It is clearly characterised by large mandibles, larger than in any other species in the complex. The median projection of T6 is widely rounded convexly, without apical emargination, and projects beyond the lateral projections (not projecting in *T. hakkariensis* sp. n. with which it may occur in the same region). T7 is parallel-sided or slightly broadened at apex and with a truncated apex (tapered with truncated apex in *T. hakkariensis* sp. n.).

The relative width of the mandible in relation to the clypeus length (index clypeus length / mandible width) is significantly higher in *T. maxima* (mean: 0.92±0.059 mm) than in the other species of the complex. It overlaps to some degree with *T. balcanica* sp. n. (mean: 1.03±0.052 mm), which is, however, the smallest species of the group (and *T. maxima* the largest), so that the combination of both characters allows an unambiguous species distinction. The clypeus of *T. maxima* is emarginate, and the apical margin crenulated with 8-11 rounded tubercles, and it shares this character with *T. balcanica* sp. n., *T. hakkariensis* sp. n., and *T. verhoeffi*. The emargination is usually shallower in the latter two species.

The genal area has a large yellow maculation which is broad at the upper end and narrow at the lower end and extends from the top of the eye to its lower end.

The median projection of T6 reaches beyond the lateral projections (Figs 7, 15) (in *T. hakkariensis* sp. n. this projection extends at most to the level of the lateral projections, and in *T. pubescens* the median projection has at its apex a small emargination or is at least truncated). The surface of T6 is shining, with fine punctures widely scattered especially in the centre of both sides; the punctures are often separated by a few puncture diameter. *Trachusa balcanica* sp. n. and *T. hakkariensis* sp. n. are similar with an on average only slightly denser punctation, while the punctation in *T. pubescens* and *T. verhoeffi* is noticeably coarser and denser. The punctures in these two species are usually subcontiguous.

####### Variability.

Seven males examined from Turkey (including the type specimen) have a relatively narrow transverse yellow band on the vertex, broken in the middle and clearly separated from the yellow maculation on the genal area. In just one of these specimens, the yellow band is merged with the yellow maculation on the genal area. One specimen (from Konya in Turkey) has two small yellow spots between the lateral yellow bands on T2, a character which is often also present in *T. pubescens* (see Fig. 11D). The mesepisternum is black in all specimens. The Armenian population is distinguished from the Turkish population by the unified yellow colouration on gena and vertex. Three of the six males examined have a yellow mesepisternal spot, while it is black in the others. Only one specimen is available from the central Iranian population, and this is the largest specimen of all the *T. pubescens* s. l. examined. Already in the initial PCA, this specimen proved to be different from all other individuals of the complex (Fig. 2, see above). It has a large yellow spot on mesepisternum and the vertex has scattered, short hairs (longer and denser in the other populations). While the PCA places the specimens of these three populations (Armenia, Iran, Turkey) in different clusters (Fig. 5), this difference does not become evident in the Discriminant Analysis (DA) (Fig. 6).

####### Distribution.

The distribution of *T. maxima* extends in the west from the northern slopes of the Taurus Mountains in Turkey to Ankara in Inner Anatolia in the north. It was also found in the Turkish south-eastern provinces of Kahraman Maraş and Adıyaman. Separated, possibly isolated populations are present in Armenia and central Iran (Isfahan). While some morphometric and colouration differences exist between the Turkish, Armenian and Iranian populations, the material is not comprehensive enough to justify the assignment to different taxa, for example to subspecies.

####### Flower preference.

In Armenia, the species was found visiting the large yellow flowers of *Phlomis* sp. (M.K.).

####### Remark.

The type locality is the Pisidian Taurus, where it was collected in 1928 (ZMB). Another male and a female were collected there at the same time (cMAV, ZMB). According to Friese (1931) the collector was the speleologist P. Weirather (not noted on specimen labels), and his collection activities and travel itineraries (Pretner 2011) point to the year 1929. The exact type locality could not be identified but it is likely that it is situated in the area around Isparta.

###### Trachusa pubescens

Taxon classificationAnimaliaHymenopteraMegachilidae

(Morawitz, 1872)

Figs 11 , 12 , 14 , 15

Anthidium pubescens Morawitz 1872: 59–60 [Note: While the date of publication is given in many publications as 1873, it has now been accepted as 27.vi.1872. See Pesenko and Astafurova (2003)].
Anthidium pubescens Morawitz (1874).

####### Lectotype.

Male, Derbent, Dagestan, Russia (ZISP). Photographs provided by Yu. Astafurova.

The species was described on the basis of two males collected in “Hab. in Caucaso, Derbent” (photographs of original labels in Proshchalykinet al. 2017). There are two males in ZISP from this locality, which corresponds to the original description of Morawitz (1872, 1874). One of these males was designated as lectotype by Proshchalykin and Astafurova in 2016 (Proshchalykin et al. 2017).

####### Material examined.

Females have been assigned to *T. pubescens* on the basis of the location of the collection area (as long as no other species of the species group are known to occur in this region: Crimea, southern Greece, northern Iran) or because they were collected together with males which were positively identified as *T. pubescens* (Turkey). **GREECE**: 2♀, 6♂, Alt-Korinth (ancient Corinth), Peloponnes, 03.06.1964, M. Schwarz leg. (6ex. cMS, 2ex. cMAV); 1♀, same location, 21.05.1964, 1♀, same location, 23.05.1962, M. Schwarz leg. (cMS); 1♀, same location, 01.06.1974, M. Schwarz leg. (cMAV); 1♂, Alt-Korinth (ancient Corinth), Solomos, Peloponnes, 24.05.1964, M. Schwarz leg. (cMS); 1♂, Athens, 02.06.1962, H. Hunder leg. (cMS); 2♀, Corinth, Peloponnes, 24.05.1962, M. Schwarz leg. (1 ex. cMAV, 1 ex. cMS); 1♀, Chelmos, Kalavrita, Peloponnes, 31.05.1962, H. Hamann leg. (cMS); 2♂, Chalkis (Euboea), V.1926, Holtz leg. (ZMB); 1♂, Hellas Alt-Korinth, 21.06.1996, W. Arens leg. (cWA); 1♂, ibid., 07.06.1997, W. Arens leg. (cWA). **IRAN**: 2♂, Elburs, Polour 22 km N Ab Ali, 13.-14.7.1965, A. G. Soika & G. A. Mavromoustakis leg. (OLL); 3♀, ibid., 11.07.1965, A. G. Soika & G. A. Mavromoustakis leg. (OLL); 1♀, Tehran, 5.-8.5.1972, H. Bytinski-Salz leg. (OLL); 3♂, Elburs, Ilekah road 4 km above Pol-e Zanguleh, 2450 m, 14.7.1967, Baker leg. (SEMC, Baker collection); 1♀, 1♂, Kakan, Yasouj, 9.07.2009 (cAM). **MACEDONIA** (Former Yugoslav Republic): 1♀, 1♂, Prilep, 01.06.1965, K. Warncke leg. (OLL). **RUSSIA**: 1♂ (paralectotype), Derbent, Dagestan (ZISP). Photographs provided by Yu. Astafurova. **TURKEY**: 1♀, 3♂, Turkey („Asia Minor“), 1890 (OLL, ZMB); 1♀, 1♂, Ankara, 16.06.1934, A. Seitz leg., “Anthidium laticeps F. Mor. det. J. D. Alfken 1934“ (ZMB); 1♂, ibdid., 21.06.1934, A. Seitz leg., “Archianthidium maximum Friese Pasteels det. 1967“ (SMF); 1♂, ibid., 24.06.1934, A. Seitz leg., “A. pubescens Pasteels det. 1976“ (SMF); 1♂, ibid., 25.06.1934, A. Seitz leg., “A. laticeps / Archianthidium pubescens J. Pasteels det. 1976” (SMF); 3♂, Ankara: 40 km W of Ayas, 26.06.1998, J. Halada leg. (cMS); 5♂, Isparta: Karakuş Dağı Centr. (38°15'N, 30°39'E), 1460 m, 11.07.2006, J. Halada leg. (cMS); 2♂, Denizli: 35 km SSE Denizli (37°37'N, 29°17'E) 970 m, 05.07.2006, J. Halada leg.; 1♀, 2♂, 28 km SSE Kütahya (39°13'N, 30°08'E) 1110 m, 12.07.2006, J. Halada leg. (cMS); 3♀, 4♂, Tatvan, Van Gölü, 01.07.2000, M. Halada leg. (cMS); 2♀, 3♂, Bitlis, Nemrut Dağı (2000 m); 28.07.1986, I. Blank leg. (OLL); 2♀, 1♂, Siirt: 10 km S, 23.-24.6.1985, M. Schwarz leg. (cMS); 2♀, 1♂, Adiyaman: Gölbaşı, 21.06.1985, M. Schwarz leg. (cMS); 1♂, Hakkâri: 16 km SE Yüksekova (1700 m), 28.06.1985, M. Schwarz leg. (cMS); 1♂, Hakkâri: Sat Mountain S Varegös (2000 m), 06.08.1982, K. Warncke leg. (OLL). **TURKMENISTAN**: 1♂, West Kopet Dagh [Köpetdag], Syunt Mts., 21.06.1953, O. Kryzhanovskiy leg. (ZISP). Photographs provided by Yu. Astafurova. **UKRAINE**: 1♂, Crimea, 03.08.1937 (SIZK); 1♂, Crimea (exact location illegible), before 1908 (SIZK); 1♂, Crimea (“Tauria”; exact location illegible), 22.06.1914, V. Pliginski leg., (cMAV); 2♂, Crimea (exact location illegible), without date (cMAV); 1♂, Crimea (“Tauria”), Sevastopol, 26.06.1911, V. Pliginski leg. (cMAV); 1♂, Crimea (“Tauria”), 21.06.1909, V. Pliginski leg. (cMAV); 1♂, Crimea, Savastopol, 26.06.1986 (SIZK); 1♂, ibid., 08.07.1912, V. Pliginski leg. (cMAV); 1♂, Karadag, Crimea, 01.07.1919 (OLL); 1♀, ibid., 17.06.1923 (OLL); 1♂, ibid., 23.06.1925, Kistjakovsky leg. (SIZK); 1♂, ibid., 22.06.1929, J. Paramonum leg. (SIZK); 1♀, 1♂, Crimea, Feodossija, 17.06.1995, C. Ivanov leg. (SIZK).

####### Other material.

**TURKEY**: The following females from Turkey are attributed to this species as “Trachusa aff. pubescens”: 1♀, Ankara, 22.06.1973, K. Warncke leg. (OLL); 1♀, Ankara: Kızılcahamam; 18.06.1985, M. Schwarz leg. (cMS); 1♀, Ankara: 10 km S Ankara, 05.06.1988, K. Warncke leg. (OLL); 1♀, Konya: 10 km S Karaman, 19.06.1985, M. Schwarz leg. (cMS); 1♀, Konya: Sille; 08.06.1972, J. Heinrich leg. (SMF); 1♀, Akşehir, 07.1934, (OLL), “A. interruptum Pasteels det. 1967 / A. pubescens det. Warncke“; 2♀, Akşehir (“Ak-Chehir“), 1900, Korb leg. (OLL); 3♀, Hakkâri: 19 km S Beyetüşşebap (1200 m), 26.06.1985, M. Schwarz leg. (cMS); 1♀, Malatya: Erkenek 60 km SW Malatya (1300 m), 26.06.2000, M. Halada leg. (cMS); 1♀, Hakkâri: 16 km SE Yüksekova (1700 m), 28.06.1985, M. Schwarz leg. (cMS); 1♀, Hakkâri, Suvari-Halil-Paß östl. Beytüşşebap (2300 m), 03.08.1982, K. Warncke leg. (OLL).

####### Differential diagnosis.

The male is characterised by the following morphological features: Apical margin of clypeus straight or only slightly curved inwards and shallowly crenulated, usually without individually discernible tubercles; lateral projections of T6 subacute, apex of median projection emarginate (variable between a shallowly emarginate and a narrow V-shaped incision); punctation of T6 dense and coarse; median projection of T7 broadened toward apex. In some specimens in which the median emargination of T6 is inconspicuous or absent, the median projection of T7 is widened. For identification at least one of these two characters should hold true. Margins of yellow maculations on abdominal terga normally irregularly ragged.

####### Variability.

Four OUs were distinguished: Greece (including one from FYR of Macedonia), Crimea, Anatolia, and northern Iran. Members of the Greek and Crimean OUs are characterised by yellow maculation in the genal area and a dark vertex. The genal maculation usually extends from the top of the eye to the lower end of the eye but is sometimes irregular (margins ragged or maculations broken). Only one specimen from the Crimea has yellow on the vertex. In the Anatolian OU, however, the yellow maculation of the genal area usually extends up to the middle of the vertex and merges, or at least the lateral bands are contiguous. There is, however, some variation within populations and, for example, specimens with merged maculation and with widely separated maculations can be found together. The colour pattern of northern Iranian specimens is in general close to the Greek and Crimean OUs but one specimen with subcontiguous bands is available.

The yellow colouration on T2 in the Crimean, Greek and northern Iranian OUs is confined to lateral bands far apart from each other, whereas in the Anatolian OU the gap between the lateral bands is narrower and two small yellow spots are normally situated between them.

Specimens of the northern Iranian OU are smaller than those in the other populations. Despite a small sample size (N = 5), the Iranian specimens proved to be significantly smaller than Anatolian specimens in 22 of 27 morphological features (at least *p* < 0.05; t-test). Anatolian specimens in turn do not differ in size from Crimean and Greek specimens.

A Discriminant Analysis (DA) was carried out to find out whether the OUs attributed to *T. pubescens* s. str. are different from each other in morphometric characters. Fig. 4 shows that the specimens from Greece, Crimea, and Anatolia form three distinctive clusters. In their morphometric characters northern Iranian specimens are between the Greek and Anatolian specimens but their separation is less clear. Nevertheless, in a confusion matrix, which summarises the reclassification of the observations and enables us to see quickly the percentage of well-classified observations, all specimens (100%) of all four groups were correctly classified.

Altogether, the populations of *T. pubescens* s. str. from Greece, the Crimea, Anatolia, and northern Iran can be separated based on a set of morphological features. Northern Iranian specimens are smaller than all others. The colouration pattern of the vertex and gena enables most Anatolian specimens to be distinguished from the others, but due to variation within populations this feature is not regarded here as being of taxonomic significance. The pattern of the yellow colouration on the first three terga is on average different in the Anatolian population from the other OUs. All these features could justify giving the OUs subspecies rank. However, the holotype of *T. pubescens* could not be examined and, although the description is detailed enough to allow unambiguous species attribution within the *T. pubescens* species group, it is not detailed enough to decide whether the colouration and/or morphometric features agree with one of the OUs described here.

####### Flower preference.

No information available.

####### Distribution.

The distribution area extends from Crimea and southern Greece over Anatolia and the Caucasus to the Kopet Dagh Mountain in Turkmenistan. In the south, the distribution area extends into the Zagross Mountains of Iran. Possible hybridisation with *T. balcanica* sp. n. takes place in the F.Y.R. Macedonia.

Mocsáry (1879, 1884) describes material from Hungary with an emarginate T6. While this character alone does not allow unambiguous species identification, it may be interpreted as an indication that the distribution of *T. pubescens* may extend further north than indicated by the records presented in this paper.

###### Trachusa verhoeffi

Taxon classificationAnimaliaHymenopteraMegachilidae

(Mavromoustakis, 1955) stat. n.

Figures 11 , 12 , 14 , 15

Archianthidium pubescens (F. Mor.) , subsp. verhoeffi . – Mavromoustakis (1955: 921–922).
Anthidium pubescens ssp. verhoeffi Morawitz, 1872. – Warncke (1980).
Trachusa (Archianthidium) pubescens verhoeffi (Mavromoustakis, 1955). – stat. n.

####### Material.

Holotype: Male. **ISRAEL**: 12-14.05.1951, P. M. F. Verhoeff leg. (cMAV). – Paratypes: 1♀ “allotype”, 1♀, 1♂ paratype, same data as holotype (cMAV).

Other material examined: **ISRAEL**: 1♂, Israel, 12–14.05.1951, P. M. F. Verhoeff leg. (cMAV); 2♀, 1♂, Jerusalem, 12-14.05.1951, P. M. F. Verhoeff leg. (cMAV); 1♂, Kirjat Anawim, 07.05.1930, Bodenheimer leg. (ZMB). **JORDAN**: 2♂, Jordan valley: Dayr Alla, 27.04.1996, Mi. Halada leg. (cMS). **LEBANON**: 2♂, Donnieh: Sfiri (34°25'N, 36°03'E) 808 m, 27.05.2012, M. Kasparek leg. (cMK). **PALESTINE** (State of): 1♂, Har Gilo, 5 km SW Jerusalem, 850 m, 23.04.1989, R. Kasher leg., *Phlomis viscosa* Labiatae (SEMC). **TURKEY**: 1♂, Akyaka: Korucak (Muğla prov.), 07.05.2013, (cMK); 1♀, 2♂, Akyaka: Kıran (700 m) (Muğla prov.), 15.05.2013, (cMK); 2♂, Akyaka: Çardak (700 m) (Muğla prov.), 15.05.2013, (cMK); 1♂, Akyaka: Çardak (700 m) (Muğla prov.), 19.05.2015, (cMK); 1♂, Akyaka: Gökçe (Muğla prov.), 13.05.2010, (cMK); 1♂, Akyaka: Korucak (Muğla prov.), 07.05.2013, (cMK); 1♂, Köyceğiz (Muğla prov.), 15.06.2016, (cMK); 1♀, Antalya: Termessos 700-1000 m (37°00'N, 30°28'E), 23.-24.5.83, Aspöck, Rausch & Ressl leg. (cMS); 1♀, Termessos, 07.05.1989, W. Perraudin leg. (OLL); 1♂, Antakya, 01.06.1965, M. Schwarz leg. (cMS); 1♂, Antakya, 04.06.1965, M. Schwarz leg. (cMS); 1♀, Antakya, 06.06.1965, M. Schwarz leg. (cMS); 1♂, Antakya env., 30.04.1994, Mi. Halada leg. (cMS); 1♂, 10 km N Saimbeyli, 120 km N Adana, 12.06.1998, Ma. Halada leg. (cMS). 1♀, 1.5 km SW Yeşilova (Ula, Muğla prov.) (75 m), 18.04.2018, H. Koç, O. Özgül & M. Kasparek leg. (cMK). – **SYRIA**: 1♀, Syria, 1886, Gödl leg. (OLL) [Note: Gödl gave only “Syria” as the location for this and for other collected insect material, without further specifications. It cannot be ruled out that the specimen was collected in an area which is nowadays in Turkey or Lebanon].

####### Differential diagnosis.

**Figure 16.**
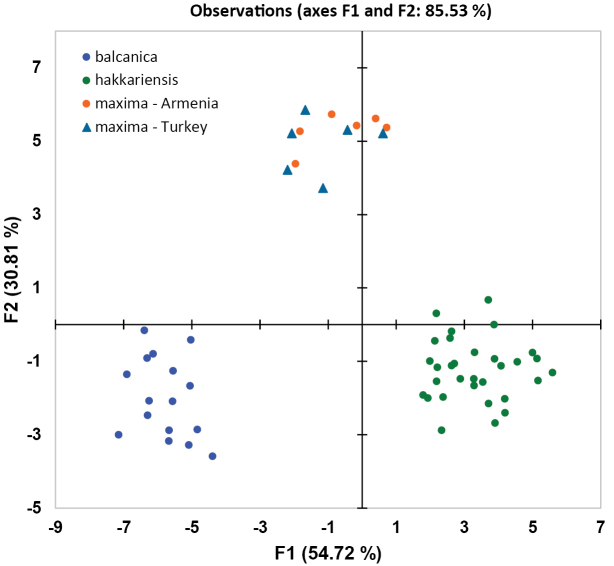
Discriminant Analysis (DA) of the 26 morphometric characters of males of *Trachusa balcanica* sp. n., *T. hakkariensis* sp. n., and *T. maxima*. Members of these OUs are characterised by an emarginate clypeus and subacute lateral projections of T6. *Trachusa maxima* is subdivided into the Armenian OU (including one specimen from central Iran) and the Anatolian OU.

**Figure 17.**
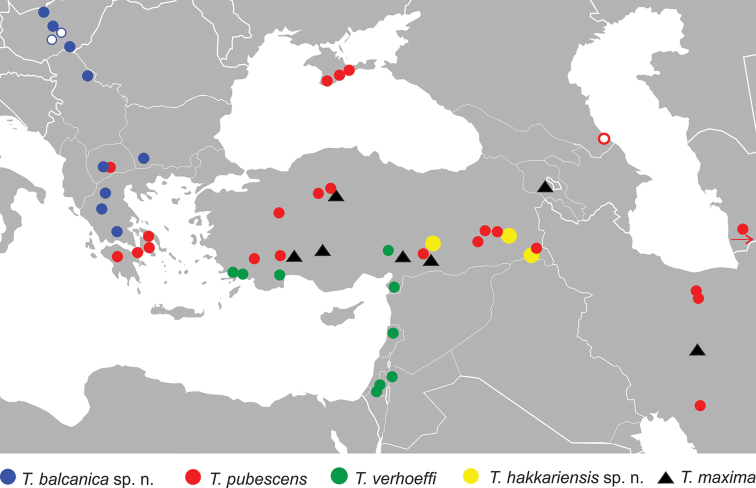
Distribution of the species of the *Trachusa pubescens* complex. The red circle shows the locus typicus of *T. pubescens* s. str. A locality of *T. pubescens* s. str. in Turkmenistan is not shown as it is beyond the limits of the map.

Males are characterised by a widely rounded median projection on T6 (apical emargination absent) combined with a short, obtuse and rounded process on each side (acute or subacute in all other species) (Figs 16, 17). Punctation on T6 slightly finer than in *T. pubescens*, but clearly coarser than in *T. hakkariensis* sp. n., *T. maxima* and *T. balcanica* sp. n. Median projection of T7 relatively broad, more or less parallel-sided (no significant apical thickening as in *T. pubescens* s. str. or apical tapering as in *T. hakkariensis* sp. n.). The apex of the clypeus is shallowly emarginate (on average slightly shallower than in *T. balcanica* sp. n., *T. maxima* and *T. hakkariensis* sp. n.) and crenulated with approximately 8-11 denticles. The abdominal T1 and T2 have a very broad transverse yellow stripe on each side not reaching the middle; T3 similar, but often extending almost to the middle (subcontiguous); T4 and T5 with broad yellow bands, broken in the middle or with a basal notch.

####### Variability.

Males from the Levantine coastal area (from the Turkish provinces of Hatay over Lebanon to Israel, Palestine, and Jordan) are much richer in yellow than those from SW Turkey. The yellow maculation extends from the lower end of one eye over the vertex to the lower end of the other eye, only narrowly broken on vertex in some specimens. In the specimens from SW Turkey, this yellow colouration is reduced to some remnants on genae and vertex. While there is great individual variation in the extent of the yellow, the yellow on the genae and vertex are never merged. Also the extent of the yellow on the scutum is much reduced in SW Turkish specimens: Levantine specimens have a broad yellow L-shaped anterolateral band on scutum and sometimes also some inconspicuous yellow spots on axillae. This yellow maculation is much reduced in SW Turkish specimens and often entirely absent. Although not apparent to the naked eye, specimens of the Levantine OU proved to be larger than those of the SW Turkish OU. From 26 morphometric measurements examined, the Levantine OU is on average larger in 24 of them. In nine of them, the size differences are significant (at least *p* <0.05, t-test).

The colouration pattern of a male from the central Taurus Mountains (Saimbeyli, Turkey) coincides with the pattern of the SW Turkish OU including the absence of yellow colouration on the scutum. On T6, the lateral projections are shallow and rounded as is typical for *T. verhoeffi*, but the apex of the median projection has a shallow emargination as is typical for *T. pubescens*. This is the only specimen with such an emargination among the 20 males examined of this taxon. Additionally, the punctation of T6 is coarser than in the other specimens and in this character also resembles *T. pubescens*.

####### Distribution.

Mavromoustakis (1955) thought that the distribution of this taxon was confined to the Levantine coastal area (Israel, Palestine, Lebanon). Warncke (1980) supposed that it extends to the Amanus Mountains in southern Turkey. The new records show that the distribution actually extends along the entire Mediterranean coastal area of Turkey and in Muğla province reaches the border with the Aegean region.

####### Flower preference.

Mavromoustakis (1955) records *Phlomis viscosa* (Lamiaceae) for Israel and Lebanon, and R. Kasher (as per collection label) found it in the West Bank (State of Palestine) on the same flowers. Kasparek (2017a) collected it in Lebanon at *Ph. chrysophylla*. Both *Phlomis* species are very similar. *Ph. viscosa* occurs along the Levantine coast up to southern Turkey (Hatay – Adana region), whereas *Ph. chrysophylla* is native to Lebanon, Syria and Israel/Palestine, i.e. not reaching so far north. In SW Turkey, Kasparek (unpubl.) collected it on Phlomis cf. fruticosa.

## Conclusions and discussion

The examination of more than 200 specimens of *T. pubescens* s. l. showed that this is actually a complex of at least five species. This includes two species which had previously been assigned as “variants” or subspecies and are elevated here to the species level. All species are clearly defined by a combination of colouration and morphometric features. They maintain either phenotypic integrity in sympatry, or, in the case of geographic replacement species, their contact zone consists of an abrupt transition with little phenotypic intergradation. Below the species level, morphometric and/or colour differences were also found between some OUs (populations), and it cannot be ruled out that some of them actually represent subspecies or even distinct species.

### Species concept

An important criterion for the assignment to species level was whether males can be clearly distinguished from the males of all other taxa by the combination of two or three functionally independent characters (Helbig et al. 2002), for example colouration plus shape of T6 or shape of clypeus plus shape of T6. Actually or potentially covarying characters, such as the extent of yellow colouration on the genae and vertex are not regarded as independent. Size differences or differences in the extent of yellow colouration alone were not taken as a basis for species assignment. For this reason, the OUs of *T. verhoeffi* in SW Turkey and the Levant were not given different taxonomic status despite some distinctive characters. Additionally the OU Armenia and the OU Anatolia of *T. maxima* are not assigned to different taxa despite differences in body size and colouration. In these cases, the evolutionary distinctness of the OUs at the species-level is doubtful and their assignment to different species would put the future integrity of the taxonomic status at risk. In *T. pubescens*, the OUs in Greece, Crimea, Anatolia, and Iran are assigned to the same species although differences are found between them both in colouration and morphometrics. Here we are faced with the dilemma that all these OUs correspond with the description of the species, but a finer classification cannot be made as the type material was not examined, and we therefore do not know which (if any) of the OUs corresponds exactly with the original name-bearing material.

The characters used for classification were the shape and size of the clypeus, mandibles, terga T6 and T7, overall colouration pattern and morphometric features. With this taxonomic concept, it was only possible to distinguish males. The colour pattern of females often resembles that of males within a certain OU, but due to the absence of distinguishing structural characters, it was impossible to determine females unambiguously. Morphometric analyses were not carried out because of the much lower number of females available (60 females compared to 148 males).

### Flower preferences

*Trachusa verhoeffi* and *T. maxima* are known to have strong preferences for visiting the large yellow flowers of various species of *Phlomis*. Species identified include *Ph. chrysophylla*, *Ph. grandiflora*, *Ph. nissolii*, Phlomis cf. fruticosa, and *Ph. viscosa* (Güler et al. 2014, Kasparek 2017a, Kasparek, unpubl.). Some of these plant species are very similar and difficult to distinguish and we cannot be sure whether the species has always been identified correctly. The flowers of *Phlomis* are arranged in whorls which encircle the stems. The flower consists of an upper and a lower lip (“Labiatae”) and the upper lip is hood-shaped and laterally compressed. *Trachusa balcanica* sp. n., the smallest species of the complex, is the only species which was observed visiting other plants and these include *Genista* (yellow flowers) and *Stachys germanica* (pink or pinkish-purple) (Friese 1898; Mocsáry 1884). Also a record on *Scabiosa ochroleuca* (whitish yellow flowers) from Moldova probably refers to this species. While *Genista* has pea-shaped flowers, the five-lobed corolla of *Stachys* has a “hood” formed by the top lobe, and *Scabiosa* has clusters of flowers (inflorescences) in the form of heads, with each head containing many small florets.

Müller (1996) showed in a pollen analyses that more than 99% of the pollen sampled from in excess of 30 specimens of *Trachusa pubescens* s. l. comes from Labiatae. His specimens were sampled over a large portion of the range of the *T. pubescens* complex. Özbek and van der Zanden (1993) mention *T. pubescens* s. l. visiting *Onobrychis viciifolia* (purple pea-shaped flowers) in eastern Turkey. It is not clear to which taxon of the *T. pubescens* complex this observation can be attributed to. According to Grace (2010), *T. pubescens* s. l. is an oligolege of Labiatae and therefore flower visit records to the Asteraceae, including *Carduus* and *Centaurea*, will refer to nectar gathering by either sex.

In Anthidiini, the females of the species studied so far are polyandrous, and polyandry is combined with male territoriality (Paxton 2005). In the case of *Anthidium*, the defended territories are the females’ food ﬂowers (Severinghaus et al. 1981). The data presented here suggest that the evolution of the *Trachusa pubescens* complex is closely related to their flower preferences. Flowers of the genus *Phlomis*, which may have played a key role, are native to Asia, southern Europe, and northern Africa and comprise approximately 113 species (IPNI 2012, Mathiesen 2006).

### Distributional relationships

The distribution of all species of the complex is given in Fig. 17. *Trachusa balcanica* sp. n. and *T. verhoeffi* have distribution areas which do not overlap with any of the other species of the group and can thus be characterised as allospecies. In contrast, the distribution areas of the other three species, *T. pubescens*, *T. maxima*, and *T. hakkariensis* sp. n. overlap to some extent and these species co-exist at least to some degree in sympatry. While two or three of these species have been found together in the same region, they have so far never been found together at exactly the same location. It is suggested that species divergence went in parallel with ecological differentiation. Niche partitioning such as flower preference is a mechanism which may be invoked to explain this.

*Trachusa pubescens* has the widest distribution, extending from the Balkan Peninsula and the Crimea Peninsula over Anatolia and the northern slopes of the Greater Caucasus (Dagestan, Russia) to the Elburs Mountains in northern Iran and the Kopet Dagh Mountain in Turkmenistan. In the Balkans, the border between the distribution of *T. pubescens* and *T. balcanica* sp. n. is sharp; both species can easily be distinguished, usually on the basis of their size and colouration alone without considering detailed morphological features. In the contact area, in the FYR of Macedonia, some specimens with intermediate characters were found. It is thought that a secondary contact zone exists there, with some hybridisation occurring there.

In Inner and Northern Anatolia, the distribution of *T. pubescens* overlaps with the distribution of *T. maxima*; both species were collected in Ankara province. However, according to the collection labels the species have never been collected together and it may well be that this is due to different ecological requirements.

*Trachusa verhoeffi* occurs along the Mediterranean coast from the southern Aegean region to the southern Levant. Its distribution area is clearly separated from those of *T. maxima* and *T. hakkariensis* sp. n. which occur north of the Taurus mountain ridge. One male collected in the central Taurus Mountains shows some characters which are close to *T. pubescens*. A final appraisal of the taxonomic status and possible hybridisation cannot be made on the basis of this single individual.

*Trachusa maxima* shows a disjunct distribution: it occurs in Inner Anatolia, south-eastern Anatolia, Armenia and central Iran. It is not known whether this scattered distribution pattern is merely the result of a lack of material. Differences between these three populations were found in terms of morphology, morphometry and colouration. They are, however, not strong enough to justify the assignment to different species. From central Iran, a single male is available, and this is by far the largest individual examined of the entire *T. pubescens* complex. More material is needed to show whether it is justified to give this OU a separate taxonomic status.

*Trachusa hakkariensis* sp. n. has a relatively narrow distribution area in eastern and south-eastern Turkey. It has been found in the same region as *T. pubescens* but never together at the same location. *Trachusa maxima* also lives in areas close to *T. hakkariensis* sp. n. but was never found together with it.

Mocsáry (1884) mentions material in the Hungarian Natural History Museum from Constantine in Algeria, which is far outside the known range of *T. pubescens* s. l. This material could not be traced (Zoltán Vas, pers. comm.) and it is suggested that it should be ignored.

### Dentition

*Trachusa pubescens* s. l. has a large apical and a smaller subapical tooth, followed by two teeth which are often merged or reduced to an edentate tooth ridge (Figs 14A, C, E). As even fresh individuals often have straight tooth ridges, a straight tooth ridge is certainly not merely the result of abrasion. In approximately 45% of all males examined, the subapical tooth is completely or almost entirely absent (see e.g., Figs 14B, D). Such a high proportion of broken teeth is unusual and raises the question of its function. It may be speculated that this tooth has a special role, e.g. in opening the walls of the brood cells.

### Colouration of tergum 6

The colouration of tergum 6 is highly variable in all species of the complex. While the median projection and the lateral teeth are always black, and also a small black v-shaped maculation is always present on the proximal side, the extent of the yellow colouration shows high variation: yellow maculation may be completely absent (resulting in an entirely black T6), may be confined to a small median spot on the surface of T6 or to two spots (one on each side), or may cover most of T6. On average, there are more specimens with entirely black T6 or with only small yellow maculation in *T. pubescens* s. str. than in the other species of the complex. Within the species, specimens with black or almost black T6 are more frequent in material from Ukraine and Greece than from Anatolia and Iran. Despite interspecific differences in the frequency pattern, this character apparently does not have taxonomic relevance.

### Melanism

A semi-melanistic individual of *T. balcanica* sp. n. was found. It had all the yellow colouration much reduced on the head, meso- and metasoma, and had black maculation also on the hind tibiae, which are normally completely yellow. As the habitus, general structure, and body measurements fall completely within the range of *T. balcanica* sp. n., it is thought that this individual belongs to this species and not to a hitherto undescribed taxon. Melanistic or partly melanistic bees have been found in several bee species and may form a certain component of a population. For example, Sheffield et al. (2011) regularly found both pale and melanistic forms in three species of *Megachile* in Canada and Alaska. In the Western Palaearctic, melanism is well known in several species of *Bombus* (e.g. Schmiedeknecht 1907, Pekkarinen and Teräs 1986) and the proportion of melanistic *Bombus* has increased recently in Finland (Södermann 1999). The phenomenon of melanism has been thought to be related to unfavourable environmental conditions in early spring (Södermann 1999). For *Andrena albihirta*, Landham (1974) found a melanism rate of up to 36 percent in twelve populations in the Rocky Mountains and suggested that black colouration functions to lengthen the active period of the species by absorption of solar heat even in reduced sunlight. Nemésio and Martins (2013) found specimens of the Neotropical orchid bee *Euglossa carolina* in which the metallic integument is replaced by black colouration, indicating a reversion in the evolution of the colouration of the integument.

### Identification key (males)

**Table:** 

1	Apical margin of clypeus straight or almost straight, shallowly crenulated with hardly distinguishable tubercles (Fig. 14E); apices of penis valves straight (Fig. 10) [SE Europe over Caucasus to Iran and Turkmenistan]	***T. pubescens* s. str.**
–	Apical margin of clypeus impressed in the middle, forming a wide emargination; margin crenulated with 8-11 rounded tubercles (Fig. 14A–D); apices of penis valves hooked	**2**
2	Lateral projections of T6 rounded (Fig. 7) [Mediterranean coast]	***T. verhoeffi* stat. n.**
–	Lateral projections of T6 subacute or acute (Fig. 7)	**3**
3	Median projection of T6 extending beyond lateral projections (Fig. 7); T7 parallel-sided or widened apically (Fig. 8)	**4**
–	Median projection of T6 not extending beyond lateral teeth (Fig. 7); T7 usually tapering towards apex (Fig. 8); small to medium-sized species [Eastern and south-eastern Turkey]	***T. hakkariensis* sp. n.**
4	Mandible large (Fig. 14D), mandibular width at base of teeth at least as long as clypeus length; large yellow maculation in genal area extending from upper to lower end of eye; large species [Anatolia and Iran]	***T. maxima* stat. n.**
–	Mandible smaller (Figs 14A–C, E), mandibular width at base of teeth less than clypeus length; yellow maculation on genal area confined to its upper half or rarely slightly beyond; yellow stripe on vertex usually separated from yellow genal maculation; small species [Balkans]	***T. balcanica* sp. n.**

## Supplementary Material

XML Treatment for Trachusa balcanica

XML Treatment for Trachusa hakkariensis

XML Treatment for Trachusa maxima

XML Treatment for Trachusa pubescens

XML Treatment for Trachusa verhoeffi
